# Changes in US Clinician Waivers to Prescribe Buprenorphine Management for Opioid Use Disorder During the COVID-19 Pandemic and After Relaxation of Training Requirements

**DOI:** 10.1001/jamanetworkopen.2022.5996

**Published:** 2022-05-12

**Authors:** Joanne Spetz, Laurie Hailer, Caryl Gay, Matthew Tierney, Laura Schmidt, Bethany Phoenix, Susan Chapman

**Affiliations:** 1 Philip R. Lee Institute for Health Policy Studies, University of California, San Francisco; 2 Healthforce Center at UCSF, University of California, San Francisco; 3 Department of Family and Community Medicine, University of California, San Francisco; 4 Department of Social and Behavioral Sciences, University of California, San Francisco; 5 Department of Community Health Systems, University of California, San Francisco; 6 Quantgal LLC, Fairfax, California; 7 Department of Family Health Nursing, University of California, San Francisco; 8 Department of Humanities and Social Sciences, University of California, San Francisco

## Abstract

This case series examines numbers of US clinicians receiving waivers from the Drug Enforcement Administration allowing them to prescribe buprenorphine for opioid use disorder before and during the COVID-19 pandemic.

## Introduction

The COVID-19 pandemic worsened the opioid overdose crisis.^1^ Buprenorphine management for opioid use disorder (OUD) reduces overdose risk and can be offered in office-based settings or via telehealth. Federal regulations require that clinicians complete training and obtain a waiver from the Drug Enforcement Administration (DEA) to prescribe buprenorphine.^2^ To increase buprenorphine access during the COVID-19 pandemic, federal and state regulations were relaxed to allow greater use of telehealth.^3^ Additionally, starting in April 2021, new guidelines allow clinicians to submit a Notice of Intent application to treat 30 or fewer patients without training; training is required for larger patient panels.^4^ This study examines the numbers of clinicians with waivers before and during the pandemic using national data.

## Methods

This secondary data analysis was considered exempt by the University of California San Francisco Committee for Human Research and the requirement for institutional review board approval and informed consent was waived. This study followed the relevant portions of the reporting guideline for case series.

We obtained quarterly DEA registrant files from the second quarter (Q2) of 2018 (ending June 30) through quarter 4 (Q4) of 2021 (ending December 31, 2021). Data included all registered clinicians with waivers, the number of patients each clinician was authorized to concurrently treat (30, 100, or 275 patients), and clinician type (ie, physician, advanced practice nurse [APN], or physician assistant). We used a case series study approach to compare quarterly changes in numbers of clinicians with waivers pre- vs postpandemic and after the relaxation of training requirements. We defined the prepandemic period as 8 quarters from 2018 Q2 through 2020 Q1 and the pandemic period as 7 quarters starting 2020 Q2. Stata MP version 15.1 (StataCorp) was used to prepare the DEA files for analysis and tabulate the quarterly numbers of clinicians with waivers by panel size. Excel (Microsoft) was used to calculate total treatment capacity if all clinicians prescribed the maximum permitted by their waivers, quarterly changes, and descriptive statistics regarding changes

## Results

**Figure 1.**
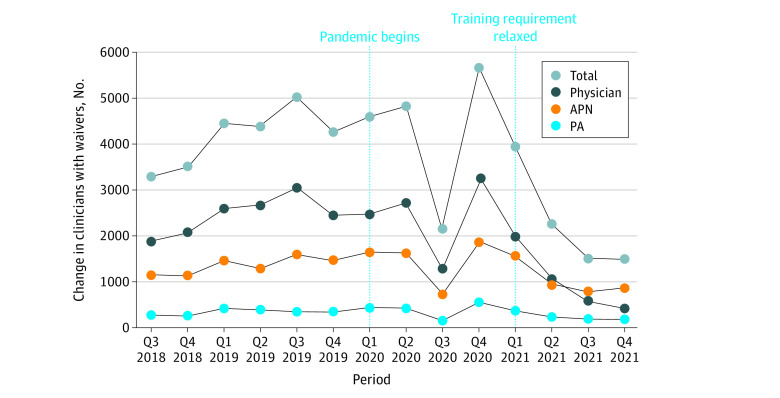
Net Quarterly Change in Number of Waivered Clinicians, Q3 of 2018 to Q4 of 2021 Q indicates quarter.

In Q2 of 2018, 47 912 clinicians had waivers to prescribe buprenorphine, including 40 405 physicians (84.3%), 5937 APNs (12.4%), and 1570 PAs (3.3%). In Q4 of 2021, there were 99 481 clinicians with waivers, including 69 089 physicians (69.4%), 24 127 APNs (24.3%), and 6265 PAs (6.3%). Before the pandemic, the number of waivers grew at a mean (SD) of 4234 (608.2) per quarter compared with 3133 (1685.8) per quarter during the pandemic (Figure 1). Compared with the prepandemic period, the mean (SD) quarterly growth in waivers dropped for all clinician types during the pandemic period (physicians, 2475 [389.2] vs 1622 [1095.2]; APNs, 1398 [202.2] vs 1200 [476.9]; and PAs, 360 [72.8] vs 310 [145]).

**Figure 2.**
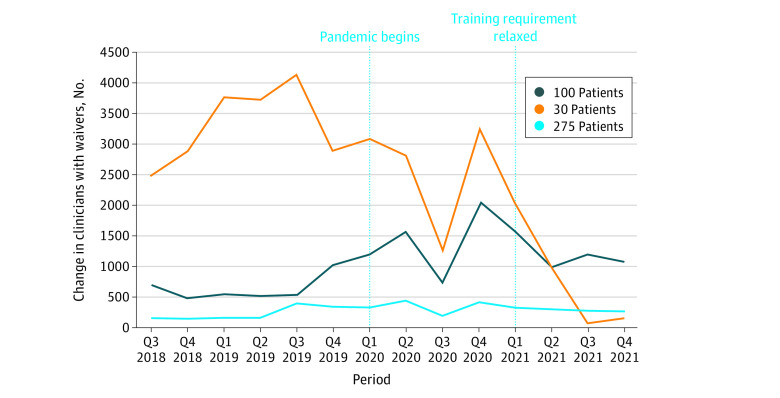
Net Quarterly Change in Number of Waivered Clinicians, by Number of Patients Authorized, Q3 of 2018 to Q4 of 2021 Q indicates quarter.

Changes in waiver growth varied by authorized concurrent panel size (Figure 2). During the prepandemic period, mean (SD) quarterly growth in 30-patient waivers was 3287 (594.8) compared with 1501 (1234.4) during the pandemic. Between Q3 and Q4 of 2021, the net 30-patient waiver growth was only 157 clinicians. In contrast, the mean quarterly growth for 100-patient waivers increased from a mean (SD) of 709 (298.1) in the prepandemic period to 1316 during the pandemic, and the mean (SD) quarterly growth of 275 patient waivers increased from 238 (103.4) in the prepandemic period to 316 (88.7) during the pandemic.

In Q1 of 2018, waivers authorized treatment for up to 3.08 million patients nationally. By Q1 of 2020, this had risen to 4.73 million patients, resulting from the mean (SD) quarterly growth of 234 851 (52 116) patients. In Q4 of 2021, there was capacity to treat 6.57 million patients concurrently; the mean quarterly growth was 263 609 (82 306) during the pandemic period.

## Discussion

Findings in this study suggest that growth in the number of clinicians with waivers to prescribe buprenorphine slowed during the pandemic. The relaxation of training requirements for 30-patient waivers in April 2021 did not mitigate this. However, the total treatment capacity has continued to rise, largely because of clinicians who already had waivers shifted to 100-patient and 275-patient waivers.

Our results are limited by registrant data that did not indicate whether clinicians were prescribing buprenorphine at all or to the maximum authorized level. Thus, the data likely overestimated treatment capacity and delivery.

The growth of the workforce providing buprenorphine treatment for OUD is increasingly important amidst long-standing shortages of buprenorphine prescribers and rising opioid overdose deaths. Expansion in the number of prescribers and total treatment capacity, alongside resources and policies to facilitate buprenorphine prescription, is necessary to extend this therapy’s reach.^5^ Efforts should be redoubled to increase recruitment of new prescribers. Pandemic-focused policies, such as expanded telehealth authority and relaxation of counseling requirements, should be extended to increase access to OUD treatment.^6^
