# Database Coding Errors

**DOI:** 10.1001/jamanetworkopen.2023.27991

**Published:** 2023-07-27

In the Research Letter, “Changes in US Clinician Waivers to Prescribe Buprenorphine Management for Opioid Use Disorder During the COVID-19 Pandemic and After Relaxation of Training Requirements,”^1^ published in *JAMA Network Open* on May 12, 2022, coding errors in the database used to determine the numbers of clinicians with waivers for prescribing at the 30-patient level resulted in publication of incorrect numbers. The correct numbers of clinicians at the 30-patient level for the second, third, and fourth quarters of 2021 were substantially higher (2223, 3610, and 3398, respectively) than the originally reported numbers (989, 54, and 157 respectively). The corrected findings indicate that the number of waivers dropped for all clinician types in quarter 3 of 2020 but then rebounded and remained similar to the prepandemic period by quarter 4 of 2021. Corrections have been made to the Results and Discussion sections and to Figures 1 and 2.
